# NLRP3 Inflammasome Simultaneously Involved in Autophagy and Phagocytosis of THP-1 Cells to Clear Aged Erythrocytes

**DOI:** 10.1155/2022/1481154

**Published:** 2022-09-30

**Authors:** Qin Li, Fengyong Zhao, Jiamin Zhang, Ying Yang, Zhonghui Guo, Chen Wang, Qixiu Yang, Ying Sun, Ziyan Zhu

**Affiliations:** Shanghai Blood Center, No. 1191, Hongqiao Rd., Changning, Shanghai 200051, China

## Abstract

Autophagy and phagocytosis are two important processes that capture and digest materials found in cellular interiors and exteriors, respectively. Aged red blood cells (RBCs) are cleared by phagocytes in vivo. We focused on determining whether autophagy occurs after phagocytes swallow sunset erythrocytes, and whether the degree of autophagy is related to scavenging ability of phagocytes to erythrocytes. In addition, the ability of NLR family pyrin domain containing protein 3 (NLRP3) inflammasome to regulate erythrocyte clearance by phagocytes and its association with autophagy-related protein 16-like protein 1 (ATG16L1) are confirmed. We constructed a stable and low-NLRP3 expression THP-1 cell line using CRISPR/Cas9 technology. The analysis of erythrocyte clearance and autophagy of THP-1 cells with low NLRP3 expression showed that autophagy changes together when THP-1 engulfs aged RBCs. The occurrence of autophagy was dominated by microtubule-associated protein 1A/1B-light chain 3- (LC3-) associated phagocytosis accompanied by canonical autophagy. A negative correlation exists between the clearance of RBCs by THP-1 cells and the degree of autophagy. Downregulating the expression of NLRP3 in THP-1 cells can simultaneously inhibit the scavenging ability of THP-1 to erythrocytes and the degree of autophagy. In addition, the autophagy inhibitor bafilomycin A1 (BafA1) can enhance the phagocytosis ability of THP-1 to erythrocytes and promote the NLRP3 activation in THP-1 cells, while the autophagy inducer rapamycin inhibits the phagocytosis ability of THP-1 to RBCs and downregulates the NLRP3 activation. Results showed that autophagy and phagocytosis may be dynamic balance processes that can provide sufficient nutrition and energy to cells. Choosing NLRP3 as a target may regulate the phagocytic ability and the degree of autophagy in the meantime. These findings may be a potential strategy for regulating the clearance rate of phagocytes to aged RBCs and the secretion of proinflammatory cytokines to ensure transfusion safety.

## 1. Introduction

Red blood cells (RBCs) are senescent during in vivo circulation or in vitro storage. The aged RBCs are cleared by several phagocytic cells, such as monocytes, macrophages, and Kupffer cells [1, 2]. Two different mechanisms of senescent erythrocyte clearance called unopsonized and opsonized phagocytoses are controlled by CD47-signal regulatory protein *α* (SIRP*α*) and immunoglobulin G- (IgG-) Fc gamma receptor (Fc*γ*R) signaling pathways, respectively [1, 3].

The elimination of aged RBCs in the body will lead to a series of downstream reactions, such as phagocytes secreting additional proinflammatory cytokines. Hod et al. [4] revealed that amounts of proinflammatory cytokines, such as interleukin- (IL-) 6, monocyte chemoattractant protein-1 (MCP-1), interferon gamma (IFN-*γ*), and tumor necrosis factor alpha (TNF-*α*), in the mouse plasma increase when subjects are infused with mouse-aged RBCs. Our previous work demonstrated that the NLR family pyrin domain containing protein 3 (NLRP3) inflammasome is activated, and the secretion of proinflammatory cytokines, such as IL-1*β*, is upregulated when phorbol-12-myristate-13-acetate- (PMA-) induced THP-1 cells clear aged RBCs through signaling pathways of either CD47-SIRP*α* or IgG Fc*γ*R [5]. These phenomena suggested that phagocyte clearance of aged RBCs may demonstrate important physiological and pathological significance.

Several studies have shown that phagocytosis plays critical roles in immunity by regulating cytokine production and releasing of inflammasome activation and clearance of invading pathogens [5–7]. Lysosomes are involved in the degradation of ingested materials in the phagocyte process. The degree and results of phagocytosis will affect cell nutrition recovery, energy metabolism, cell survival, and immune regulation [8–10]. Notably, autophagy is another important system that presents similar processes and functions [8, 11–14]. Therefore, several researchers have explored the relationship between autophagy and phagocytosis. Studies on macrophage cell lines have shown that autophagy activity contributes to the phagocytic ability and degradation through toll-like receptor (TLR) signaling pathways. The activation of TLRs, especially TLR4 and TLR7, will induce autophagy in macrophages when macrophages ingest pathogens, dead cells, or misfolded protein [15–18]. This phenomenon is termed microtubule-associated protein 1A/1B-light chain 3- (LC3-) associated phagocytosis (LAP) and considered a novel noncanonical form of autophagy [19–21]. Studies on another phagocytic immune cell called microglia revealed that the degradation of extracellular *β*-amyloid fibrils by autophagy in microglia induces the NLRP3 inflammasome activation [22], and inhibiting the class I phosphoinositide 3-kinase (PI3K) pathway can both suppress the autophagy and phagocytic capacity of microglia [23].

These findings indicated that autophagy and phagocytosis are closely related, and many pathways can possibly regulate the two mechanisms. Our previous work showed that the NLRP3 inflammasome is activated when macrophage cell lines engulf aged RBCs [5]. Meanwhile, Cho et al. [22] determined that NLRP3 inflammasome is also activated when microglia autophagy degrades *β*-amyloid fibrils. In addition, NLRP3 inflammasome is closely related to autophagy-related protein 16-like protein 1 (ATG16L1) [24, 25]. The autophagy inducer rapamycin inhibits NLRP3 inflammasome activation through autophagy induction [26]. These findings suggested that NLRP3 inflammasome is simultaneously involved in autophagy and phagocytosis and may be a potential regulatory site for controlling autophagy and phagocytosis. This work is aimed at clarifying whether autophagy (LAP or canonical autophagy) occurs simultaneously when macrophage lines clear aged RBCs and determine whether a rule exists between autophagy and macrophage clearance rate of aged RBCs when autophagy occurs. We select NLRP3 as the breakthrough point, construct the THP-1 cell line with stable and low expression of NLRP3 via CRISPR/Cas9 technology, and analyze the clearance rate of THP-1 on aged RBCs and its autophagy.

## 2. Materials and Methods

### 2.1. THP-1 Cell Culture and Construction of Low-Expression Strain of NLRP3 Inflammasome

The human monocytic leukemia cell line THP-1 (from the cell bank of Chinese Academy of Sciences) was cultured in RPMI-1640 medium (Gibco) supplemented with 10 IU/mL of penicillin, 10 *μ*g/mL of streptomycin (Life Technologies), and with or without 10% fetal bovine serum (Gibco). All cells were incubated in 5% CO_2_ at 37°C.

Cells were transferred to 24-well cell culture plates when the cell concentration reached 80% and then cultured at 37°C in 5% CO_2_ until the cell concentration reached approximately 50%. THP-1 cells were subsequently transferred using CRISPR/Cas9 for NLRP3 lentivirus vector at multiplicity of infection (MOI) = 40. The CRISPR/Cas9 editing system for human NLRP3 inflammasome was designed according to the sequence from NCBI NM_001079821. Three schemes were designed for exons: 9 (ID3, Dharmacon GSGH11935-247552931), 7 (ID4, Dharmacon GSGH11935-247629609), and 5 (ID5, Dharmacon GSGH11935-247663883) of the NLRP3 gene. An empty vector marked shNC (Dharmacon GSGC11953) was also transferred to THP-1 as a nontargeting control. All products were obtained from the PerkinElmer Company Horizon™. Fresh and complete RPMI-1640 medium was replaced after incubation at 37°C for 24 hours. RPMI-1640 containing 3 *μ*g/mL of puromycin was replaced after 48 h of viral infection. The medium containing puromycin was changed every 2 to 3 days until the wells were full of cells.

RNA of THP-1 was extracted using TRIzol (Life Technologies) method. Samples were adjusted to the same concentration after reverse transcription to cDNA using the PrimeScript^TM^ II Kit (6210A, Takara, Japan). The NLRP3 gene expression was determined via QRT-PCR. The glyceraldehyde-3-phosphate dehydrogenase (GAPDH) gene was used as the internal control. The relative amount of NLRP3 gene expression was calculated using the 2 − ΔΔCt method. The primer sequence and PCR procedure are shown in supplemental data. In addition, the expression of NLRP3 inflammasome was detected using immunoblotting with anti-NLRP3 (AG-20B-0014-C100, AdipoGen) at the protein level. GAPDH (TA802519, OriGene Technologies) was used as the internal control.

The minimum NLRP3 expression THP-1 cell line was culture expanded and cryopreserved according to experimental needs. The expression of NLRP3 was evaluated again after cell recovery and passage using QRT-PCR and immunoblot.

### 2.2. Collection and Treatment of Blood Samples

Whole blood samples were collected from healthy Rh blood group system D antigen (RhD) positive donors at the Shanghai Blood Center. All procedures were reviewed and approved by the Shanghai Blood Center Medical Ethical Committee (approval number SBC-IRB-2019-18).

Blood samples were treated using the method in our previous work [5]. Erythrocytes were washed three times with a physiological saline solution (PBS). RBCs were then divided into three aliquots. One aliquot was incubated at 42°C for 2 h to prepare aged erythrocytes. Another was sensitized with the IgG anti-D antibody (Shenxing, Shanghai Hemo-Pharmaceutical & Biological Inc., Shanghai, China). The last aliquot remained untreated and then marked “untreated RBCs.”

### 2.3. mRFP-GFP-LC3 Lentivirus Vector Transfer THP-1

Double-labeling monomeric red fluorescent protein- (mRFP-) green fluorescent protein- (GFP-) LC3 lentivirus vector (Sangon Biotech) was transferred to THP-1 cells using the same method as the transfection of CRISPR/Cas9 for the NLRP3 lentivirus vector. Wild-type, shNC, and minimum NLRP3 expression THP-1 cell stains were all transferred to double-labeling vectors.

### 2.4. THP-1 Cell Phagocytosis Assay

Three groups of 9 × 10^5^ THP-1 cells (wild-type, shNC, and minimum NLRP3 expression THP-1 cells; THP-1 cells transfected with mRFP-GFP-LC3 double-labeling vectors were not used in the test because detecting RFP and GFP fluorescence signals in the phagocytosis assay was unnecessary) were cultured in each well of a six-well plate containing RPMI-1640 medium supplemented with 10 IU/mL of penicillin, 10 *μ*g/mL of streptomycin, and 100 ng/mL of PMA (P1585, Sigma–Aldrich) without fetal bovine serum. Cells were then incubated in 5% CO_2_ at 37°C for 48 h to induce the state of starvation.

The phagocytosis assay was measured using Meinderts' method [27] with some modifications. Briefly, untreated, aged, and IgG-opsonized RBCs were resuspended in serum-free medium and then resuspended to 1 × 10^7^/mL. RBCs were stained with 1,1′-dioctadecyl-3,3,3′,3′-tetramethylindodicarbocyanine (DiD) lipophilic membrane dye (2157164, Life Technologies), and the final concentration was 1 *μ*L/mL. The RBC-DiD mixture was incubated at 37°C for 10 min. RBCs were washed three times with RPMI-1640 medium. RBCs were incubated with PMA-treated THP-1 cells for 4 h at 37°C (10 : 1 ratio, 9 × 10^6^ RBCs vs. 9 × 10^5^ THP-1). Bafilomycin A1 (BafA1) (A601116, Sangon Biotech), an autophagy inhibitor, and rapamycin (V900930, Sigma–Aldrich), an autophagy inducer, were added to the system for prereaction for 1 h at a final concentration of 100 and 800 nmol/L, respectively. Dye-labeled RBCs were then added to the mixture and reacted for 4 h at 37°C. Supernatant was removed and RBC lysis solution was added at the end of incubation to remove nonphagocytized RBCs. THP-1 cells were digested using 0.25% trypsin, and cell suspension was prepared in RPMI-1640 medium. Phagocytosis was determined using flow cytometry (BD FACSVerse™). Allophycocyanin (APC) channel was utilized to collect fluorescence signals. Ten thousand THP-1 cells were counted in each test. The RBC phagocytic rate was calculated as the number of APC signal-positive THP-1 cells/10,000 THP-1 cells. Each group repeated the test three times.

### 2.5. Detection of Autophagy Markers and NLRP3 Expression after Phagocytosis Assay via Immunoblot Assay

THP-1 cells coincubated with different RBCs were collected. Wild-type, shNC, and low-NLRP3 expression THP-1 cells were triggered with 800 nmol/L of rapamycin to create the autophagy positive control. THP-1 cells were lysed in a radio immunoprecipitation assay (RIPA) buffer (C500005, Sangon Biotech). The expression of autophagy markers on THP-1 was detected using immunoblotting with anti-p62 (5114) and anti-LC3 (2775, Cell Signaling Technology). Anti-NLRP3 (AG-20B-0014-C100, AdipoGen) and antigasdermin D (GSDMD) (HA601046, Hangzhou HuaAn Biotechnology Co., Ltd.) were applied to determine the activation of NLRP3 inflammasome. GAPDH (TA802519, OriGene Technologies) was used as internal control. Each blot was duplicated three times. BandScan software was utilized after development to analyze and record the gray value of the strip. Relative amounts of p62/GAPDH, LC3II/GAPDH, and LC3II/LC3I were adopted to estimate the autophagy situation of THP-1 cells, while those of NLRP3/GAPDH and GSDMD/GAPDH were used to evaluate the activation of NLRP3 inflammasome.

### 2.6. Enzyme-Linked Immunosorbent Assay (ELISA)

The concentrations of IL-1*β* and cysteinyl aspartate specific proteinase 1 (caspase-1) in the supernatant of cell culture were detected by IL-1*β* ELISA kit (ELH-IL1*β*, RayBiotech) and caspase-1 simplestep ELISA kit (ab219633, Abcam), respectively. The tested culture supernatants were collected after THP-1 cells and RBCs coincubation. The results were gained through microplate reader (Spark 10 M, Tecan).

### 2.7. THP-1 Autophagy Showed by Microscopes

Wild-type, shNC, and minimum NLRP3 expression THP-1 cells transferred with the mRFP-GFP-LC3 lentivirus vector were coincubated with different RBCs and rapamycin with or without BafA1 for 4 h at 37°C. THP-1 cells were then collected, and RBCs were removed using RBC lysis solution. THP-1 cells were stained with 4,6-diamidino-2-phenylindole (DAPI) dye (C1002, Biyuntian). Images were acquired using a fluorescence inverted microscope (BX53, Olympus, Japan). Emission wavelengths of 358, 488, and 532 nm were selected to collect DAPI, GFP, and RFP fluorescence signals, respectively. Nuclei of THP-1 cells were displayed under the 358 nm emission wavelength after staining with the DAPI dye. Stably expressed mRFP-GFP-LC3 fusion protein of THP-1 cells will display a green or red fluorescent signal in the microscope field depending on the wavelength of excitation light. Fluorescent spots may be red or yellow after merging pictures from two different optical signals. According to the manufacturer's instructions, yellow spots represent autophagosomes and red spots represent autolysosomes. If red spots are dominant in cells, then autophagic flux increases. Meanwhile, if yellow spots are dominant, then the autophagic flux decreases. The increase and decrease of autophagic flux represent the respective increase and decrease of autophagy in the detection system. Images were further analyzed via the software ImageJ to count the number of cells. The degree of autophagy was assessed via the proportion of red fluorescent cells in the total number of cells.

Images were also taken using confocal (DeltaVision, GE Healthcare Life Sciences) and transmission electron (FEI Tecnai G20 TWIN, FEI company) microscopes.

### 2.8. Statistics

Data were presented as mean ± SE. Statistical analyses were conducted using Student's t-test with SPSS 16.0 (Chicago, IL, USA). Statistical significance was considered at the 95% level (*p* < 0.05).

## 3. Results

### 3.1. Interference of Exon 9 of the NLRP3 Gene Effectively Downregulates the NLRP3 Expression

**Figure 1:**
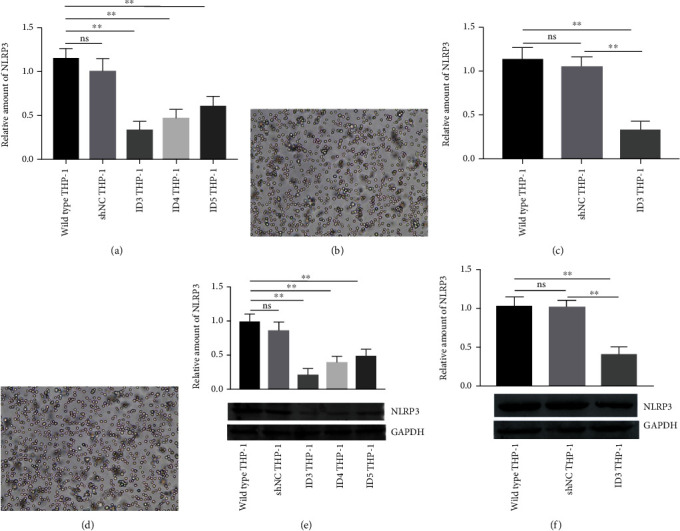
Results of CRISPR/Cas9 editing for NLRP3 in THP-1 cells and the stable transfectant THP-1 cell line. (a) Use of three programs to edit exons 9 (ID3), 7 (ID4), and 5 (ID5) of the NLRP3 gene in THP-1 cells. The shNC indicated nontargeting control. The expression of NLRP3 was determined via QRT-PCR. All three schemes can significantly downregulate the NLRP3 gene expression, and ID3 obtained the optimal regulation results. ID3 THP-1 was used in the follow-up study. (b)–(d) Microscopic examination results (100×) of THP-1 cells stably transfected with shNC and ID3 vector. Cells demonstrated normal morphology and ability to proliferate and generate. (c) NLRP3 expression in the stable transfectant ID3 THP-1 cell line tested via QRT-PCR. The expression of NLRP3 in ID3 THP-1 was still stable and low after more than three passages. (e) Evaluation of the regulatory effect of three gene-editing schemes on NLRP3 via immunoblot. ID3 showed optimal ability to downregulate the NLRP3 expression. (f) NLRP3 expression in the stable transfectant ID3 THP-1 cell line tested using immunoblot. The result was similar to that obtained via QRT-PCR. ^∗∗^*p* < 0.01, ns: not significant.

The QRT-PCR results showed that CRISPR/Cas9 editing the exon 9 of the NLRP3 gene can significantly downregulate the NLRP3 mRNA expression by 72.0%, while editing exons 7 and 5 can reduce NLRP3 mRNA expression levels by 59.4% and 48.1%, respectively, in repeated screening experiments (Figure 1(a)). Relative amounts of NLRP3 obtained via the immunoblot showed that the expression of NLRP3 protein decreases by 79.7%, 59.7%, and 52.6% after gene-editing exons 9, 7, and 5 of the NLRP3 gene, respectively (Figure 1(e)). The results of the immunoblot and QRT-PCR show similar trends. The stable THP-1 cell line with the minimum expression of NLRP3 was constructed by editing exon 9 of the NLRP3 gene in the follow-up work. The expression of NLRP3 mRNA in the THP-1 interference group was approximately 31.1% of that in the untreated group after several passages, while that in the THP-1 transfected with shNC vector was 33.6% (Figure 1(c)). The relevant results obtained through the immunoblot were 39.06% and 39.98% (Figure 1(f)). The QRT-PCR and immunoblot results demonstrated that the edited exon 9 of the NLRP3 gene of THP-1 cells still present low NLRP3 expression after multiple cell passages. The THP-1 cell line with stable and low expression of NLRP3 was named ID3 THP-1 and used in subsequent experiments.

### 3.2. Clearance Rate of Different Treated RBCs

IgG-opsonized RBCs exhibited the maximum tendency for clearance by THP-1, whereas the phagocytosis rate of untreated RBCs showed the minimum tendency with or without editing of NLRP3 genes of THP-1 cells. Phagocytic rates of untreated, 42°C-incubated, and IgG-opsonized RBCs in the three groups of THP-1 cells are listed in Table 1.

### 3.3. Downregulation of NLRP3 in THP-1 Cells Inhibits the Phagocytic Ability of THP-1 to Erythrocytes

Phagocytosis rates of untreated, 42°C-incubated, and IgG-opsonized RBCs in ID3 THP-1 cells were significantly lower than those of wild-type and shNC THP-1 cells. Erythrocyte clearance between wild-type and shNC THP-1 cells demonstrated no significant difference (see Table 1). Compared with those of wild-type THP-1 cells, phagocytosis capacities of ID3 THP-1 cells to untreated, 42°C-incubated, and IgG-opsonized RBCs decreased by 63.0%, 42.5%, and 48.3%, respectively.

### 3.4. Engulf Ability of THP-1 Cells to Erythrocytes Is Enhanced by BafA1

The phagocytosis rate of both wild-type and gene-edited THP-1 cells to erythrocytes increased when the autophagy inhibitor BafA1 was added to the THP-1 and erythrocyte coincubation system (see Table 2). Although ID3 THP-1 cells showed the lowest phagocytosis rate to erythrocytes, the increased erythrocyte clearance rate of ID3 THP-1 cells stimulated by BafA1 was significantly higher than that of wild-type and shNC THP-1 cells compared with the results of non-BafA1 groups (see Table 2).

### 3.5. Phagocytosis Ability of THP-1 Cells to RBCs Is Inhibited by Rapamycin

Phagocytosis rates of three groups of THP-1 cells to RBCs were all inhibited when rapamycin was present in the culture mixture (see Table 3). Notably, ID3 THP-1 cells still showed the minimum phagocytosis rate to RBCs, and the decreased erythrocyte clearance rate of ID3 THP-1 cells stimulated by rapamycin was also significantly higher than that of wild-type and shNC THP-1 cells compared with the results of groups without rapamycin (see Table 3).

### 3.6. Autophagy Degree Changes Simultaneously when THP-1 Scavenges Erythrocytes

**Figure 2:**
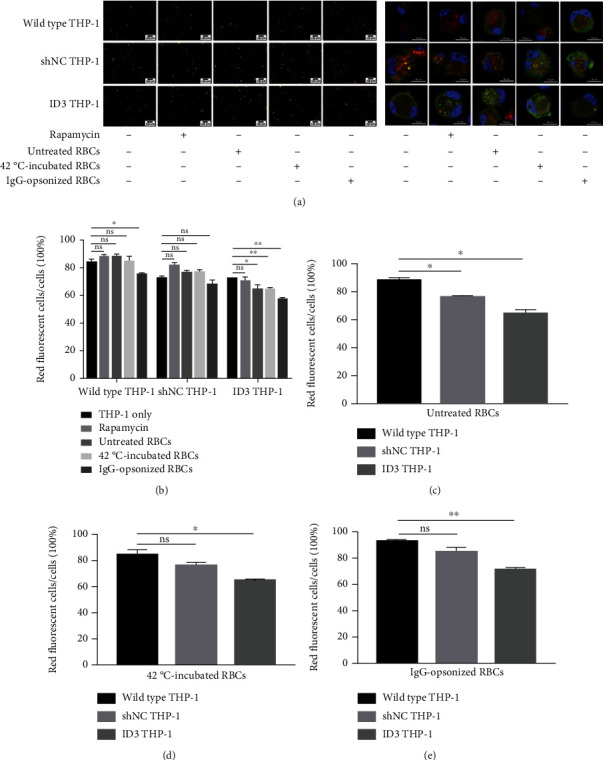
Results of autophagic flux in different THP-1 cells cocultured with three groups of RBCs. (a) Merge photograph of THP-1 cells stably expresses the mRFP-GFP-LC3 fusion protein after different stimulations. The left column shows the imaging results of the fluorescence inverted microscope (200×), whereas the right column presents the results of the confocal microscope (1,000×). The rapamycin group was the autophagy positive control. Each image of the confocal microscope showed one to two THP-1 cells. Compared with wild-type and shNC THP-1 cells, ID3 THP-1 cells demonstrated additional yellow dots when ID3 THP-1 received the same stimulation or engulfed the same treated RBCs. A large number of THP-1 cells can be displayed in the field of the fluorescence inverted microscope. Additional yellow cells were displayed in ID3 THP-1 cell groups. (b) Histograms of red cell rates of different RBCs engulfed by three groups of THP-1 cells. (c)–(e) Histograms of red cell rates of three different THP-1 cells to the same RBCs. ^∗^*p* < 0.05, ^∗∗^*p* < 0.01, ns: not significant.

The results of the confocal microscope showed that red dots are dominant in THP-1 cells when wild-type, shNC, and ID3 THP-1 cells are in the state of starvation or induced by rapamycin. Yellow dots were dominant in all the three tested THP-1 cells after engulfing IgG-opsonized RBCs. THP-1 cells showed limited red dots and additional yellow dots with untreated or 42°C-incubated RBCs (Figure 2(a)). We need additional cells in the field of vision to analyze the fluorescence displayed by THP-1 cells after phagocytosis of RBCs given that only a few THP-1 cells are shown in the confocal microscope vision. Therefore, we focus on images from the fluorescence inverted microscope. The number of green or red fluorescent cells changed after imaging when THP-1 cells received different stimuli. According to the calculated proportion of red fluorescence cells to the total cells in each visual field, proportions of red fluorescence cells were the minimum in the three groups of THP-1 cells when they phagocytize IgG-opsonized RBCs, while rapamycin-induced group and THP-1 without any stimulation showed an increased proportion of red cells (Figure 2(b)). The change trend of fluorescence spots obtained via the fluorescence inverted microscope was consistent with that obtained with the confocal microscope. As THP-1 cells demonstrated a high tendency to clear IgG-opsonized RBCs. The results suggested that autophagy of THP-1 cells is low when THP-1 cells engulf many RBCs. Images from the transmission electron microscope are shown in supplemental data.

**Figure 3:**
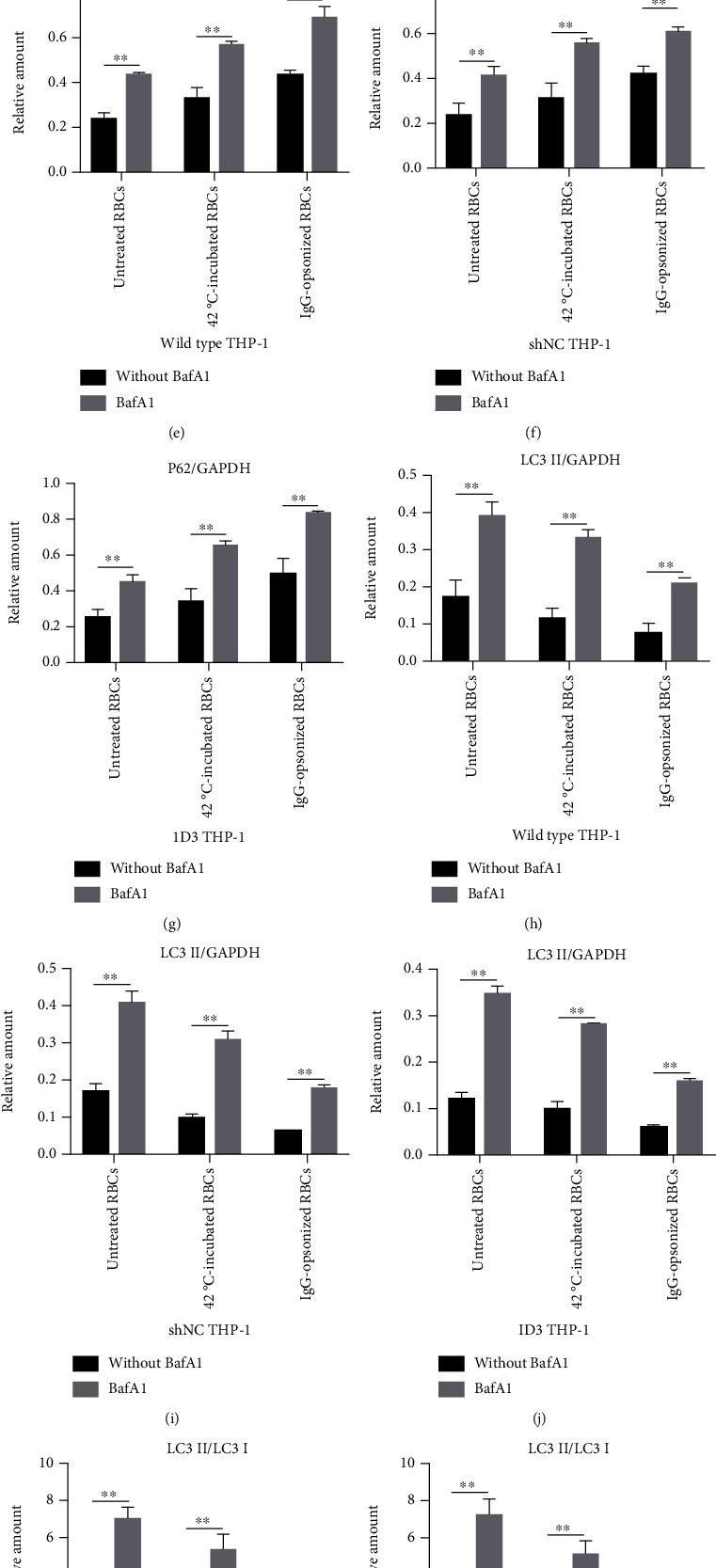
Autophagy markers determined via immunoblotting. (a) Expression of two autophagy markers, namely, p62 and LC3, in THP-1 cells after their engulfment of RBCs is determined with immunoblotting. The rapamycin group is the autophagy positive control. BafA1 is used as the autophagy inhibitor. (b)–(d) Histograms illustrating the relative amounts of p62 and LC3 II to GAPDH and those between LC3 II and LC3 I. (e)–(m) Relative amounts of p62 and LC3 II of THP-1 cells coincubated with three different RBCs with or without bafromycin. ^∗^*p* < 0.05, ^∗∗^*p* < 0.01, and ns: not significant.

Expression of autophagy markers p62 and LC3 was determined via immunoblot (Figure 3(a)). Relative amounts of p62 and LC3 II to GAPDH and those between LC3 II and LC3 I were used to analyze the autophagy of THP-1 cells. The expression of p62 decreases while that of LC3 II and the ratio between LC3 II and LC3 I increase when autophagy occurs. Histograms in Figures 3(b)–3(d) showed that the autophagy degree of THP-1 varies simultaneously when wild-type and gene-edited THP-1 cells engulf erythrocytes or are stimulated with rapamycin. The autophagy degree from high to low was rapamycin-stimulated, THP-1 only, untreated RBC, 42°C-incubated RBC, and IgG-opsonized RBC groups in the three groups of THP-1 cells. The results of the clearance rate of THP-1 cells to erythrocytes demonstrated that a high phagocytosis rate of THP-1 cells to erythrocytes indicates a low degree of autophagy of THP-1 cells. The findings suggested that the degree of autophagy of THP-1 cells is closely related to the phagocytosis rate of RBCs. If the phagocytosis rate of THP-1 to RBCs is low, then the degree of autophagy of THP-1 is high in the same group of THP-1 cells. THP-1 also presents a certain degree of autophagy when only THP-1 cells are present in the culture system. THP-1 is likely starved while autophagy occurs when PMA induces THP-1 and serum-free medium is used. The results showed that the degree of autophagy caused by starvation in THP-1 cells is lower than that induced by rapamycin but higher than that caused by phagocytosis of RBCs, especially 42°C-incubated and IgG-opsonized RBCs.

### 3.7. Autophagy of THP-1 Caused by Engulfing RBCs Is Inhibited by the Autophagy Inhibitor BafA1

**Figure 4:**
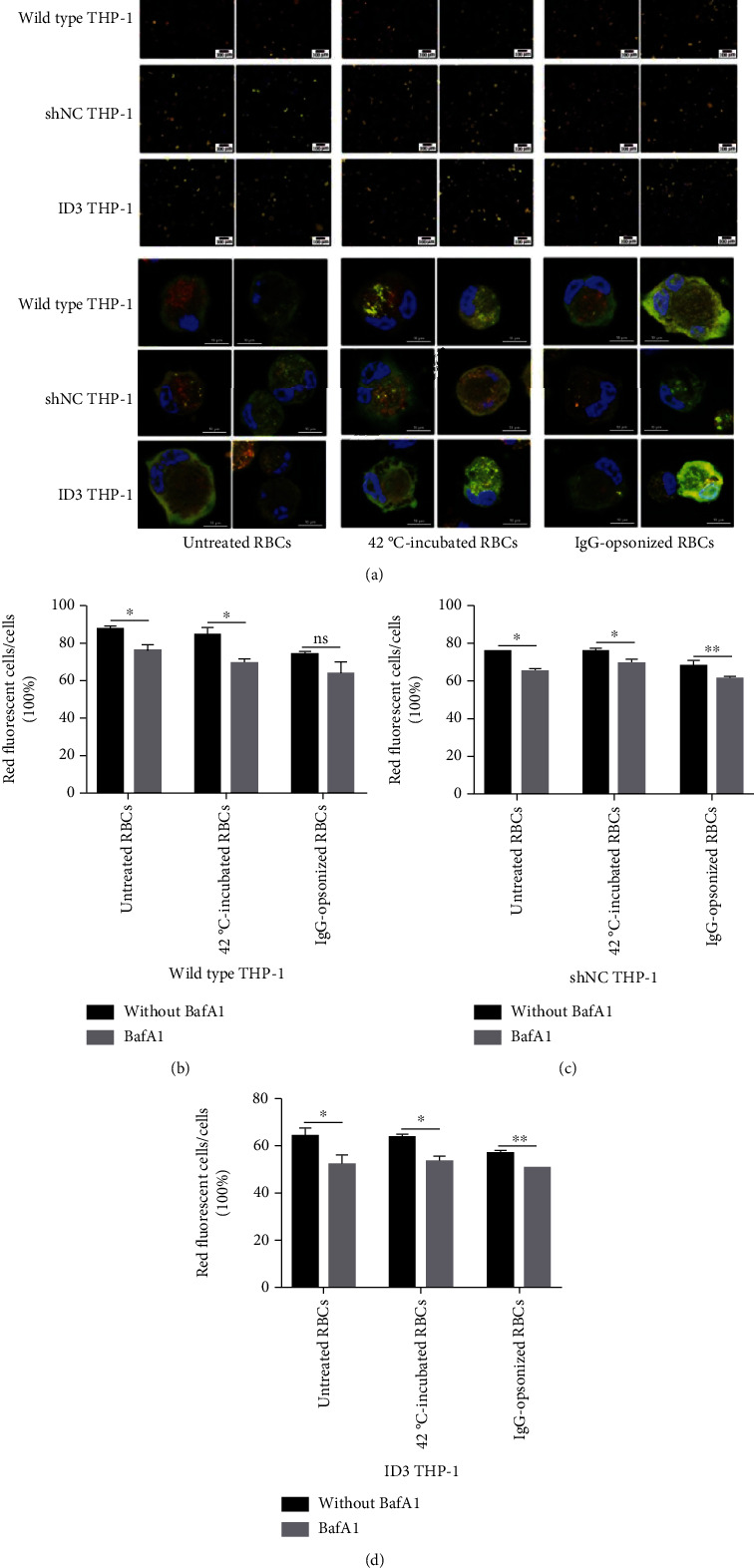
Autophagy of THP-1 caused by engulfing RBCs is inhibited by the autophagy inhibitor bafromycin. (a) Merge photograph of THP-1 cells stably expresses the mRFP-GFP-LC3 fusion protein after different stimulations. The above column shows the imaging results of the fluorescence inverted microscope (200×), whereas the below column indicates the results from the confocal microscope (1,000×). The method of image data analysis and processing is the same as that used in Figures 3(b)–3(d). (b)–(d) Histograms of red cell rates of three different THP-1 cells to the same RBCs with or without bafromycin. ^∗^*p* < 0.05, ^∗∗^*p* < 0.01, and ns: not significant.

The three kinds of THP-1 cells showed many yellow dots after coincubating with RBCs when BafA1 is present in the culture system (Figure 4(a)). The proportion of red fluorescent cells in the visual field decreased after the three groups of THP-1 cells swallow RBCs when BafA1 is present in the culture system. The downregulation effect of BafA1 on the autophagic flux caused by clearing IgG-opsonized RBCs using wild-type THP-1 cells demonstrated no statistical difference but those of the two other groups significantly downregulated. The results suggested that the autophagic flux caused by phagocytosis of erythrocytes by THP-1 cells can be downregulated with BafA1 (Figures 4(b)–4(d)).

Relative amounts of p62 and LC3 II determined through immunoblotting showed that the change trend of autophagy index of the three groups of THP-1 cells remains the same after adding BafA1 and phagocytizing erythrocytes. Relative amounts of p62 and LC3 II increased in the BafA1 addition group (Figures 3(e)–3(m)). All the findings showed that BafA1 can inhibit the autophagy of THP-1 cells caused by engulfing RBCs.

### 3.8. Low-NLRP3 Expression THP-1 Cells Show Decreased Autophagy during their Clearance of RBCs

Data obtained from the fluorescence microscope were analyzed to estimate the autophagy of the three groups of THP-1 cells after phagocytizing the same kind of RBCs. Compared with that of wild-type THP-1 cells, the proportion of red cells in the ID3 THP-1 group significantly decreased while those in wild-type and shNC THP-1 cells were nearly the same (Figures 2(c)–2(e)). The results suggested that the autophagic flux caused by phagocytosis of erythrocytes of THP-1 cells can be inhibited by downregulating the expression of NLRP3.

**Figure 5:**
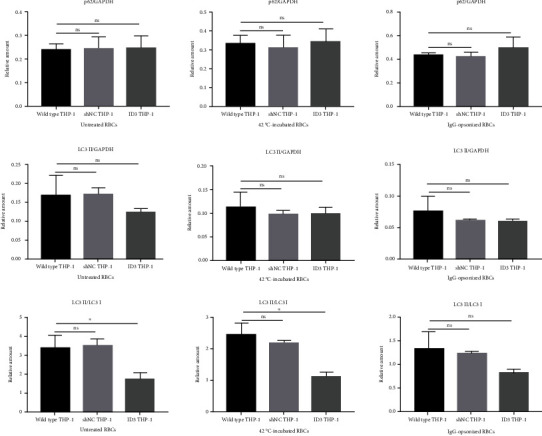
Autophagy of THP-1 caused by engulfing RBCs is inhibited by the downregulated expression of NLRP3. Three relative amounts (p62 vs. GAPDH, LC3 II vs. GAPDH, and LC3 II vs. LC3 I) were calculated according to gray values obtained in Figure 6(a). These data focus on the autophagy of wild type, shNC, and ID3 THP-1 cells after phagocytosis of the same RBCs without bafromycin. ID3 THP-1 cells showed the minimum autophagy marker regardless of the kind of RBCs they were incubated with. ^∗^*p* < 0.05, ^∗∗^*p* < 0.01, and ns: not significant.

Three relative amounts (p62 vs. GAPDH, LC3 II vs. GAPDH, and LC3 II vs. LC3 I) were calculated using the immunoblot. The expression of p62 was higher than that of wild-type and shNC THP-1 cells, and the expression of LC3 II was lower than that in wild-type and shNC THP-1 cells when ID3 THP-1 cells engulfed erythrocyte. Change trends were observed although many groups did not differ statistically (Figure 5). These phenomena combined with microscopic imaging data suggested that the downregulation of NLRP3 can inhibit autophagy of THP-1 cells caused by phagocytosis of erythrocytes.

### 3.9. Activation of NLRP3 While THP-1 Cells Engulf RBCs Scavenges Erythrocytes

**Figure 6:**
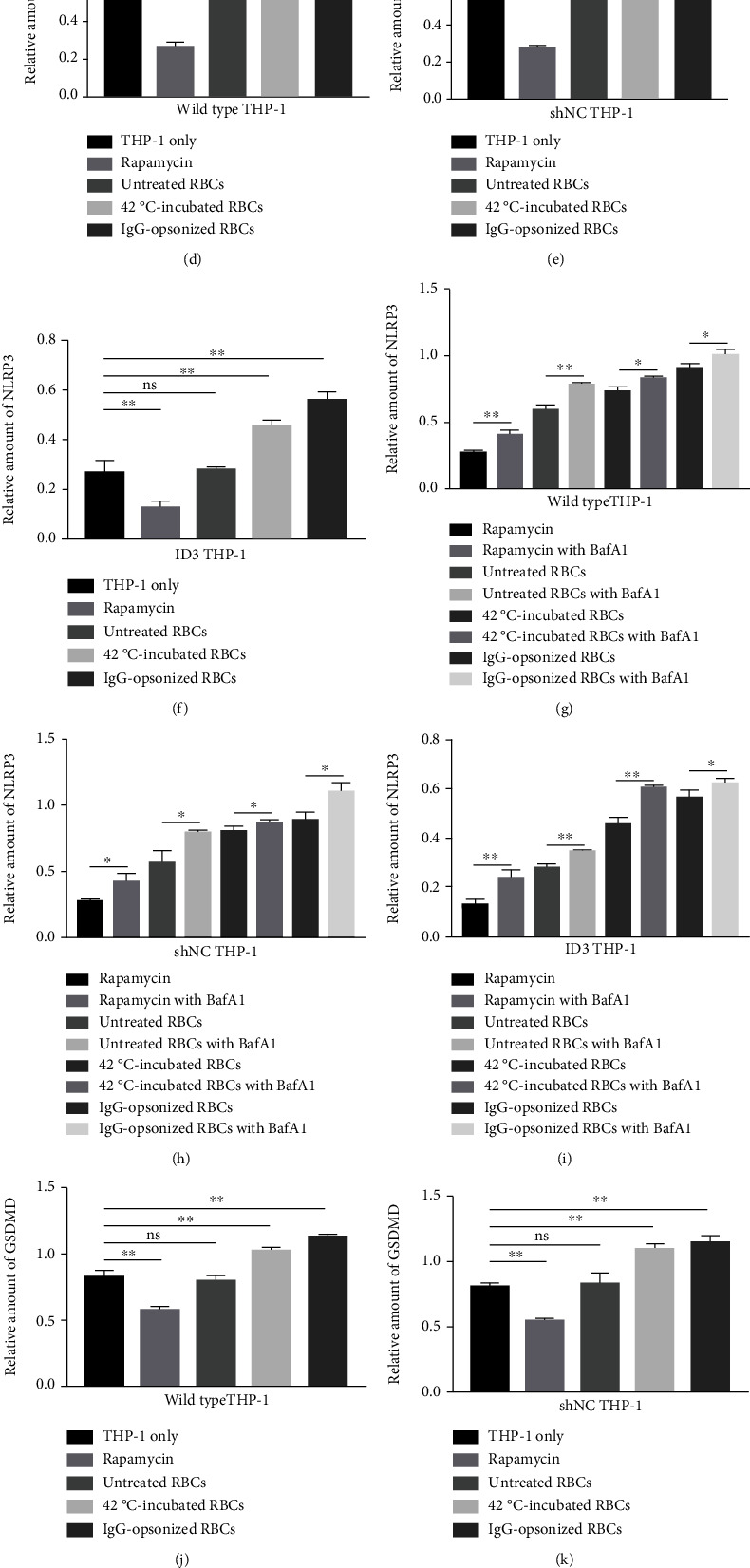
NLRP3 and GSDMD determined via immunoblotting. (a) The expressions of NLRP3 and GSDMD determined via immunoblot. (b) Histograms of the NLRP3 activation state of three different THP-1 cells after receiving the same stimulation in nine groups. (c) Relative quantity histograms of GSDMD of three different THP-1 cells after receiving the same stimulation in nine groups. (d)–(f) Histograms of the NLRP3 relative amounts of wild-type, shNC, and ID3 THP-1 cells after phagocytosis of erythrocytes. (g)–(i) Regulatory effect of BafA1 on NLRP3 expression. (j)–(l) Histograms of the GSDMD relative amounts of wild-type, shNC, and ID3 THP-1 cells after phagocytosis of RBCs. (m)–(o) Regulatory effect of BafA1 on GSDMD expression. ^∗^*p* < 0.05, ^∗∗^*p* < 0.01, and ns: not significant.

**Figure 7:**
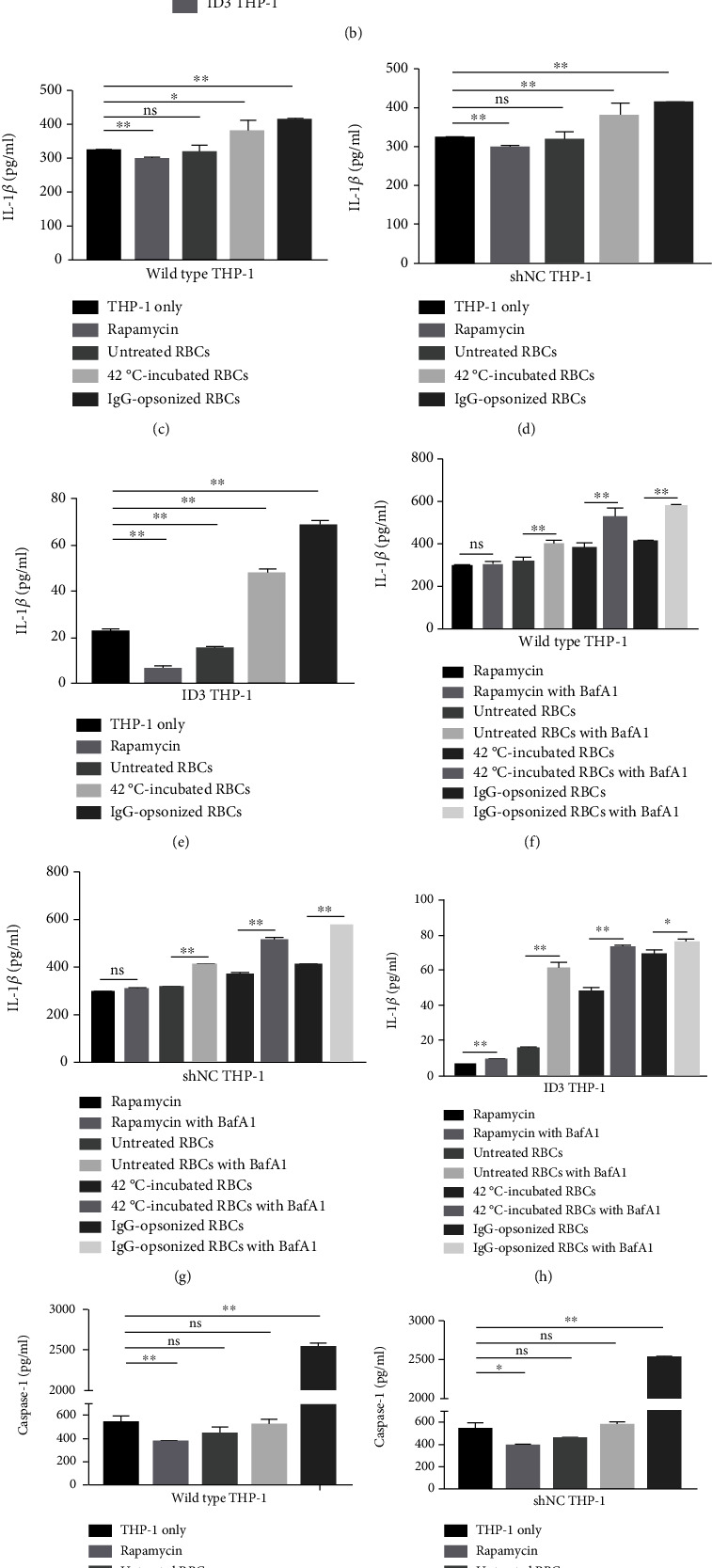
The amounts of IL-1*β* and caspase-1 detected by ELISA. (a) The concentrations of IL-1*β* of three different THP-1 cells after receiving the same stimulation in nine groups. (b) The concentrations of caspase-1 of three different THP-1 cells after receiving the same stimulation in nine groups. (c)–(e) The concentrations of IL-1*β* of wild-type, shNC, and ID3 THP-1 cells after phagocytosis of erythrocytes. (f)–(h) Regulatory effect of BafA1 on IL-1*β* secretion. (i)–(k) The concentrations of caspase-1 of wild-type, shNC, and ID3 THP-1 cells after phagocytosis of erythrocytes. (l)–(n) Regulatory effect of BafA1 on caspase-1 expression. ^∗^*p* < 0.05, ^∗∗^*p* < 0.01, and ns: not significant.

Four parameters were used to evaluate the activation of NLRP3 inflammasome. The expressions of NLRP3 and GSDMD were detected by immunoblotting (Figure 6). Gray value rates of NLRP3/GAPDH and GSDMD/GAPDH were used to analyze the activation degree of NLRP3 inflammasome in each experimental group. The concentrations of IL-1*β* and caspase-1 in the cell culture supernatant were detected by ELISA (Figure 7).

The relative quantity histograms of NLRP3 and GSDMD have similar change tendency. The expressions of NLRP3 inflammasomes and GSDMD were significantly upregulated when the three THP-1 cells, regardless of wild-type or gene-editing groups, swallowed 42°C-incubated or IgG-opsonized RBCs. The height of the NLRP3 and GSDMD relative volume histogram slightly increased although the statistical difference was insignificant after THP-1 cells phagocytized untreated RBCs compared with that of the control group without RBCs (Figures 6(d)–6(f), 6(j)–6(l)). The autophagy inducer rapamycin significantly downregulated the expression of NLRP3 and GSDMD in THP-1 cells (Figures 6(d)–6(f), 6(j)–6(l)). The expressions of NLRP3 and GSDMD were significantly upregulated in all test groups when the autophagy inhibitor BafA1 was present in the culture system (Figures 6(g)–6(i), 6(m)–6(o)). The expression of NLRP3 and GSDMD in ID3 THP-1 cells were the minimum while the difference between wild-type and shNC THP-1 cells was insignificant in the nine tested groups (Figures 6(b) and 6(c)).

The IL-1*β* and caspase-1 in ID3 THP-1 cells showed the minimum concentrations while the difference between wild-type and shNC THP-1 cells was insignificant in the nine tested groups (Figures 7(a) and 7(b)). THP-1 cells, regardless of wild-type or gene-editing groups, secreted more IL-1*β* and caspase-1 after they swallowed IgG-opsonized RBCs (Figures 7(c)–7(e), 7(i)–7(k)). The amounts of IL-1*β* and caspase-1 showed no statistical difference after THP-1 cells phagocytized untreated RBCs compared with that of the control group without RBCs (Figures 7(c)–7(e), 7(i)–7(k)). When THP-1 cells phagocytized 42°C-incubated RBCs, the secretion of IL-1*β* was significantly higher than that of the control group without RBCs (Figures 7(c)–7(e)), while the secretion of caspase-1 was not significantly changed compared with the control group (Figures 7(i)–7(k)). The autophagy inducer rapamycin significantly downregulated the ability of THP-1 cells to secrete of IL-1*β* and caspase-1 (Figures 7(c)–7(e), 7(i)–7(k)), while the autophagy inhibitor BafA1 enhanced the ability of THP-1 cells to secrete of IL-1*β* and caspase-1 (Figures 7(f)–7(h), 7(l)–7(n)).

All the results suggested that rapamycin could inhibit the activation of NLRP3 inflammasome, while BafA1 could increase the activation of NLRP3. In addition, the data also showed that the more erythrocytes engulfed by THP-1 cells, the higher activation degree of NLRP3 was displayed.

## 4. Discussion

We focused on determining whether autophagy occurs simultaneously in THP-1 cells when they clear aged erythrocytes and whether the degree of autophagy is related to the clearance rate of RBCs in the study. We constructed a stable THP-1 cell line with low expression of NLRP3 inflammasome to analyze whether NLRP3 can regulate the clearance ability of THP-1 to RBCs and whether it exerts an effect on the autophagy of THP-1 cells.

The results of our in vitro experiments revealed that the degree of autophagy of wild-type or gene-edited THP-1 cells will change when they swallow RBCs. The degree of autophagy of THP-1 cells was highly correlated with their clearance rate to erythrocytes. A high number of RBCs cleared by THP-1 cells indicates a low degree of autophagy in THP-1 cells, but the phagocytic ability of THP-1 cells to erythrocytes decreased significantly after the autophagy of THP-1 cells was stimulated by rapamycin.

Autophagy and phagocytosis were involved in cellular metabolism and feeding. Our results suggested that autophagy and phagocytosis may be a dynamic balance process that can provide sufficient nutrition and energy for cells. The degree of autophagy of THP-1 cells decreased when THP-1 cells swallow sufficient external substances, such as aged RBCs. If phagocytosis is reduced, then THP-1 cells increases autophagy to consume portions of the cell's own cytoplasm and generate essential nutrients and energy.

LAP is a novel form of noncanonical autophagy appropriately characterized in professional phagocytes, especially in macrophages [18–21, 28, 29], and commonly regulated in phagocytes to remove extracellular particles, such as apoptotic cells and pathogens. The LAP pathway shared some canonical autophagy proteins [15, 18, 30]. Our work revealed that the expression of LC3 changes during the phagocytosis of RBCs by THP-1 cells and confirmed that the LAP pathway is involved in the clearance of aged erythrocytes by THP-1 cells through the unopsonized or opsonized pathway. A variety of receptors that activate LAP, such as TLRs and immunoglobulin (Ig) receptors, have been identified [15, 18, 21, 31, 32]. IgG-opsonized RBCs combined with Ig receptors and then activated the LAP pathway. The triggering mechanism of the unopsonized clearance pathway of RBCs on the LAP pathway requires further analysis. Moreover, LAP remains unaffected when rapamycin or starvation is used [18, 32, 33]. However, the degree of autophagy changes after starving THP-1 cell-phagocytized RBCs in our work. These results suggested that both LAP and canonical autophagies of other mechanisms may occur after THP-1 scavenges erythrocytes.

p62 is an important autophagy marker that can regulate the closure of the autophagosome by simultaneously binding with LC3 proteins and ubiquitinated materials [34–36]. The results of immunoblotting showed that p62 also changes significantly (Figure 3(d)) after THP-1 cells phagocytized RBCs with different treatments. This finding suggested that p62 also plays an important role in the modulation of THP-1 cell autophagy caused by phagocytosis of erythrocytes.

We regarded THP-1 cells with low expression of NLRP3 inflammasome as the research object of this study to analyze whether NLRP3 can regulate the ability of THP-1 cells in phagocytizing RBCs and autophagy. The phagocytosis ability to erythrocytes and autophagy was downregulated in low-NLRP3 expression THP-1 cells (ID3 THP-1). Notably, although the phagocytosis ability of ID3 THP-1 cells to erythrocytes and their autophagy are both downregulated, the dynamic balance between erythrocyte clearance and autophagy still exists. Hence, the degree of autophagy reduces with the increase in number of erythrocytes phagocytized by ID3 THP-1. The mechanism of this phenomenon requires further analysis. The amounts of NLRP3, GSDMD, IL-1*β*, and caspase-1 were used to evaluate the activation of NLRP3. IL-1*β* and caspase-1 are important indicators of NLRP3 activation [37]. GSDMD belongs to the gasdermin family and serves as a specific substrate of inflammatory caspases (caspase-1, -4, -5, and -11). GSDMD is required for the release of the inflammatory cytokine IL-1*β* [38, 39]. GSDMD is also an important mark for NLRP3 activation. All the results showed that the autophagy inhibitor BafA1 can enhance the activation of NLRP3, while the autophagy inducer rapamycin inhibits the activation of NLRP3. The data also showed that the more erythrocytes engulfed by THP-1 cells, the higher activation degree of NLRP3 on THP-1 cells was displayed. The findings suggested that NLRP3 inflammasome may simultaneously play an important role in both the autophagy and phagocytosis of THP-1 cells.

Our previous work showed that pretreatment of THP-1 cells with the NLRP3 inflammasome inhibitor can downregulate the clearance of THP-1 cells to erythrocytes [5]. The RBC clearance capacity of THP-1 cells with low expression of NLRP3 achieved via CRISPR/Cas9 gene-editing was also downregulated. These results suggested that NLRP3 may be a key regulatory site for controlling the phagocytosis ability of phagocytes. ATG16L1 was required for LAP [21, 40], and Atg16L1 was closely related to the NLRP3 inflammasome. Atg16L1 can regulate the activation of NLRP3 inflammasome and secrete proinflammatory cytokines, such as IL-1*β* and IL-18 [24, 25]. We speculated that the downregulation of the NLRP3 inflammasome may negatively regulate ATG16L, inhibit the occurrence of LAP, and suppress the autophagy ability of THP-1 cells with low expression of NLRP3. An in-depth investigation is necessary to clarify this assumption. This work only focuses on the autophagy and phagocytosis of THP-1 cells with low expression of NLRP3. A follow-up study can consider the autophagy and phagocytosis of THP-1 cells with high expression of NLRP3.

Many patients undergoing blood transfusion treatment have received blood that has been stored in blood banks for a long time. The rapid clearing of sunset RBCs by phagocytes of patients will affect the effect of blood transfusion treatment. The results of in vitro experiments and the murine model showed that the secretion of proinflammatory cytokines increases when phagocytes clear aged RBCs [4, 5, 41, 42]. The normal threshold of proinflammatory cytokines was necessary to maintain organism health, and excessive proinflammatory cytokines may cause damage to the body. Our work showed that phagocytosis of erythrocytes will change the degree of autophagy of THP-1 cells, and the LAP pathway may be the key mechanism of THP-1 cell autophagy induced by phagocytosis of erythrocytes. The occurrence of LAP was accompanied by the secretion inhibition of proinflammatory cytokines, such as IL-6, IL-1*β*, and chemokine (C-X-C motif) ligand 10 (CXCL10) [43–46]. Although some studies still maintain that the relationship between autophagy and cytokine secretion is unclear [47–50], regulating the autophagy pathway may be a potential scheme for modulating the cytokine secretion. Downregulating the NLRP3 expression in phagocytes with some autophagy enhancers or by providing sufficient nutrition to phagocytes may reduce the phagocytic ability of phagocytes to aged erythrocytes and secretion of proinflammatory cytokines. A series of new strategies for regulating the clearance rate of phagocytes to aged RBCs and secreting proinflammatory cytokines may be suggested to improve the blood utilization rate and transfusion treatment effect and ensure the safety of patients.

## 5. Conclusion

The degree of autophagy of THP-1 cells and the phagocytic ability of THP-1 cells to aged RBCs are highly negatively correlated when THP-1 cells clear aged RBCs. NLRP3 inflammasome is simultaneously involved in autophagy and phagocytosis of THP-1 cells to clear aged RBCs and plays an important regulatory role. The degree of autophagy and phagocytosis of THP-1 cells to aged erythrocytes are both downregulated when suppressing the NLRP3 expression on THP-1 cells. In addition, the autophagy inhibitor BafA1 promotes the activation of the NLRP3 inflammasome in THP-1 cells, while the autophagy inducer rapamycin inhibits the activation. Note that this phenomenon still exists in low-NLRP3 expression THP-1 cells although the autophagy and phagocytosis abilities both decreased. NLRP3 inflammasome may be a potential target for regulating the clearance rate of phagocytes to aged RBCs and the secretion of proinflammatory cytokines to ensure transfusion safety.

## Figures and Tables

**Table 1:** Results of THP-1 cells engulfed RBCs.

	Phagocytosis rates of RBCs (100%)			*p* value		
	Untreated RBCs	42°C-incubated RBCs	IgG-opsonized RBCs	1	2	3
Wild-type THP-1	13.12 ± 0.34	31.56 ± 0.41	44.54 ± 0.06	^∗∗^	^∗∗^	^∗∗^
shNC THP-1	13.50 ± 0.12	31.72 ± 1.00	44.87 ± 0.40	^∗∗^	^∗∗^	^∗∗^
ID3 THP-1	4.85 ± 0.19	18.15 ± 0.68	23.02 ± 0.23	^∗∗^	^∗∗^	^∗∗^
*p* value						
4	Ns	Ns	Ns			
5	^∗∗^	^∗∗^	^∗∗^			
6	^∗∗^	^∗∗^	^∗∗^			

*p* value, 1: untreated RBCs vs. 42°C-incubated RBCs; 2: untreated RBCs vs. IgG-opsonized RBCs; 3: 42°C-incubated RBCs vs. IgG-opsonized RBCs; 4: wild-type THP-1 vs. shNC THP-1; 5: Wild-type THP-1 vs. ID3 THP-1; and 6: shNC THP-1 vs. ID3 THP-1. ^∗∗^*p* < 0.01, ns: not significant.

**(a):** 

Phagocytosis rates of RBCs (100%)									
	Untreated RBCs			42°C-incubated RBCs			IgG-opsonized RBCs		
	BafA1	Without BafA1	*p* value	BafA1	Without BafA1	*p* value	BafA1	Without BafA1	*p* value
Wild-type THP-1	17.68 ± 0.41	13.12 ± 0.34	^∗∗^	39.24 ± 0.39	31.56 ± 0.41	^∗∗^	58.40 ± 0.33	44.54 ± 0.06	^∗∗^
shNC THP-1	18.01 ± 0.36	13.50 ± 0.12	^∗∗^	39.58 ± 0.85	31.72 ± 1.00	^∗∗^	58.39 ± 0.14	44.87 ± 0.40	^∗∗^
ID3 THP-1	12.31 ± 0.34	4.85 ± 0.19	^∗∗^	26.18 ± 0.50	18.15 ± 0.68	^∗∗^	34.97 ± 0.73	23.02 ± 0.23	^∗∗^

**(b):** 

	Increased RBC clearance rate enhanced by BafA1 (100%)			*p* value		
	Wild-type THP-1	shNC THP-1	ID3 THP-1	1	2	3
Untreated RBCs	35.00 ± 6.51	33.42 ± 2.32	154.32 ± 13.91	Ns	^∗∗^	^∗∗^
42°C-incubated RBCs	24.36 ± 1.05	24.95 ± 5.23	44.38 ± 4.49	Ns	^∗∗^	^∗∗^
IgG-opsonized RBCs	31.11 ± 0.90	30.15 ± 1.48	51.90 ± 2.84	Ns	^∗∗^	^∗∗^

*p* value, 1: Wild-type THP-1 vs. shNC THP-1; 2: Wild-type THP-1 vs. ID3 THP-1; and 3: shNC THP-1 vs. ID3 THP-1. ^∗∗^*p* < 0.01, ns: not significant.

**(a):** 

Phagocytosis rates of RBCs (100%)									
	Untreated RBCs			42°C-incubated RBCs			IgG-opsonized RBCs		
	With rapamycin	Without rapamycin	*p* value	With rapamycin	Without rapamycin	*p* value	With rapamycin	Without rapamycin	*p* value
Wild-type THP-1	9.11 ± 0.34	13.12 ± 0.34	^∗∗^	23.89 ± 0.63	31.56 ± 0.41	^∗∗^	30.88 ± 0.50	44.54 ± 0.06	^∗∗^
shNC THP-1	9.17 ± 0.41	13.50 ± 0.12	^∗∗^	23.72 ± 0.54	31.72 ± 1.00	^∗∗^	30.87 ± 0.41	44.87 ± 0.40	^∗∗^
ID3 THP-1	1.85 ± 0.19	4.85 ± 0.19	^∗∗^	10.15 ± 0.54	18.15 ± 0.68	^∗∗^	12.02 ± 0.73	23.02 ± 0.23	^∗∗^

**(b):** 

	Decreased RBC clearance rate inhibited by rapamycin (100%)			*p* value		
	Wild-type THP-1	shNC THP-1	ID3 THP-1	1	2	3
Untreated RBCs	30.55 ± 0.80	32.09 ± 3.20	61.98 ± 2.54	Ns	^∗∗^	^∗∗^
42°C-incubated RBCs	24.31 ± 1.50	25.10 ± 3.32	44.03 ± 3.34	Ns	^∗∗^	^∗∗^
IgG-opsonized RBCs	30.68 ± 1.04	31.18 ± 0.31	47.78 ± 3.01	Ns	^∗∗^	^∗∗^

*p* value, 1: Wild-type THP-1 vs. shNC THP-1; 2: Wild-type THP-1 vs. ID3 THP-1; and 3: shNC THP-1 vs. ID3 THP-1. ^∗∗^*p* < 0.01, ns: not significant.

## Data Availability

All data supporting the conclusions are presented in the manuscript. Additional information will be made available by the corresponding author (https://mailto:liqin@sbc.org.cn) upon reasonable request.

## Supplementary Materials

Supplementary Materials Supplemental Methods: QRT-PCR primers and procedure for NLRP3. Table 1 Primers for NLRP3. Table 2 QRT-PCR reaction system and procedure. Supplemental Figure 1: TEM images of the structure of autophagosomes and phagosomes.
