# Supplementary material for: NLRP3 Inflammasome Simultaneously Involved in Autophagy and Phagocytosis of THP-1 Cells to Clear Aged Erythrocytes
Source: J Immunol Res. 2022 Sep 30;2022:1481154. doi: 10.1155/2022/1481154 (PMC9546708; doi:10.1155/2022/1481154)
Supplement: Supplementary Materials — Supplemental Methods: QRT-PCR primers and procedure for NLRP3. Table 1 Primers for NLRP3. Table 2 QRT-PCR reaction system and procedure. Supplemental Figure 1: TEM images of the structure of autophagosomes and phagosomes. [file 1481154.f1.pdf]

Supplemental Data

Supplemental Methods:

QRT-PCR primers and procedure for *NLRP3*

Table 1 Primers for *NLRP3*

Gene	Primer	Sequence (5'-3')	PCR Products
Homo GAPDH	Forward	TCAAGAAGGTGGTGAAGCAGG	115 bp
	Reverse	TCAAAGGTGGAGGAGTGGGT	
Homo NLRP3	Forward	GTTTGACCCCGATGATGAGC	244 bp
	Reverse	CTTGTGGATGGGTGGGTTTG	

Table 2 QRT-PCR reaction system and procedure

cDNA	4 μl
Forward Primer (10 μM)	0.4 μl
Reverse Primer (10 μM)	0.4 μl
SYBR Green Master Mix	10 μl
50×ROX Reference Dye 2	0.4 μl
H_2O	4.8 μl

Procedure:

Items	Temperature	Time	Cycles
Predenaturation	95 ℃	10 min	1
Denaturation	95 ℃	15 sec	40
Annealing extension	60 ℃	60 sec	
Melting curve	95 ℃	15 sec	1
	60 ℃	60 sec	
	95 ℃	15 sec	

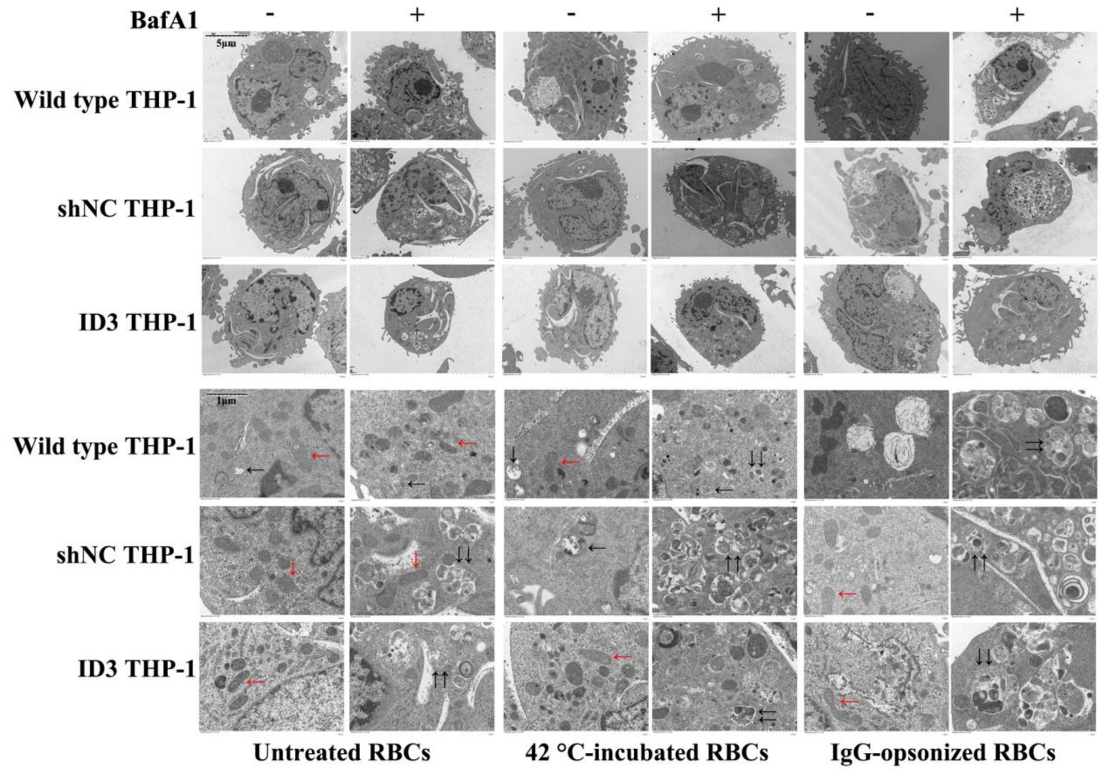

Supplemental Figure 1. TEM images of the structure of autophagosomes and phagosomes. Arrows indicate autophagosomes, double arrows indicate phagosomes, and red arrows indicate mitochondria. The above column show the imaging results of TEM (3,000×, scale=5 μm), whereas the below column depict the TEM results (12,000×, scale=1 μm). The three THP-1 cells (wild-type, shNC, and ID3 THP-1) all show additional autophagosomes under starvation and rapamycin stimulation. The number of autophagosomes in THP-1 cells decrease and the number of phagosomes increase when THP-1 cells engulf RBCs, especially 42 ℃-incubated and IgG-opsonized RBCs. Phagosomes in THP-1 cells are significantly upregulated while the number of autophagosomes significantly decrease in BafA1 preinduced groups after phagocytosis of RBCs is treated using the same method. The number of autophagosomes and phagosomes in ID3 THP-1 cells with low expression of NLRP3 inflammasome is less than that in wild-type and shNC THP-1 cells after receiving the same stimulation.
